# Effects of arecoline on proliferation of oral squamous cell carcinoma cells by dysregulating c-Myc and miR-22, directly targeting oncostatin M

**DOI:** 10.1371/journal.pone.0192009

**Published:** 2018-01-31

**Authors:** Jureeporn Chuerduangphui, Tipaya Ekalaksananan, Ponlatham Chaiyarit, Natcha Patarapadungkit, Apinya Chotiyano, Bunkerd Kongyingyoes, Supannee Promthet, Chamsai Pientong

**Affiliations:** 1 Department of Microbiology, Faculty of Medicine, Khon Kaen University, Khon Kaen, Thailand; 2 HPV & EBV and Carcinogenesis Research Group, Khon Kaen University, Khon Kaen, Thailand; 3 Department of Oral Diagnosis, Faculty of Dentistry, Khon Kaen University, Khon Kaen, Thailand; 4 Research Group of Chronic Inflammatory Oral Diseases and Systemic Diseases Associated with Oral Health, Faculty of Dentistry, Khon Kaen University, Khon Kaen, Thailand; 5 Department of Pathology, Faculty of Medicine, Khon Kaen University, Khon Kaen, Thailand; 6 Anatomical Pathology Unit, Khon Kaen Hospital, Khon Kaen, Thailand; 7 Department of Pharmacology, Faculty of Medicine, Khon Kaen University, Khon Kaen, Thailand; 8 Department of Epidemiology, Faculty of Public Health, Khon Kaen University, Khon Kaen, Thailand; 9 ASEAN Cancer Epidemiology and Prevention Research Group, Khon Kaen University, Khon Kaen, Thailand; University of South Alabama Mitchell Cancer Institute, UNITED STATES

## Abstract

Arecoline, the major alkaloid of areca nut, is known to induce oral carcinogenesis, however, its mechanism is still needed to elucidate. This study investigated the effects of arecoline on cell viability and cell-cycle progression of oral squamous cell carcinoma (OSCC) cells as well as a relevant cellular gene expression. The results showed that a low concentration of arecoline (0.025 μg/ml) increased OSCC cell viability, proportion of cells in G2/M phase and cell proliferation. Simultaneously, it induced IL-6, STAT3 and c-Myc expression. Interestingly, c-*myc* promoter activity was also induced by arecoline. MiR-22 expression in arecoline-treated OSCC cells was suppressed and comparable to an upregulated c-Myc expression. In arecoline-treated OSCC cells, oncostatin M (OSM) expression was significantly upregulated and inversely correlated with miR-22 expression. Likewise, OSM expression and its post-transcriptional activity were significantly decreased in miR-22-transfected OSCC and 293FT cells. This result demonstrated that miR-22 directly targeted OSM. Interestingly, miR-22 played an important role as a tumor suppresser on suppressing cell proliferation, migration and cell-cycle progression of OSCC cells. This result suggested the effect of arecoline to promote cell proliferation and cell-cycle progression of OSCC cells might be involved in induction of c-Myc expression and reduction of miR-22 resulting in OSM upregulation.

## Introduction

Areca nut chewing that is most frequently done in Asia, is a major risk factor for oral squamous cell carcinoma (OSCC) [1]. Arecoline is the main alkaloid in areca nut and is known to have cytotoxic, genotoxic and mutagenic properties, contributing to histologic changes and other biological consequences [2, 3]. It is likely that the effects of arecoline vary depending on cell type, individual idiosyncrasy and dose. However, little is known as yet about the various effects of arecoline.

Activation of c-Myc is a critical process in cancer development/progression [4]. Various factors can induce c-Myc expression by activation of mitogenic signaling cascades, including IL-6/STAT3 signaling cascade, etc [5]. The few studies about the effect of arecoline on c-Myc induction have been controversial.

MicroRNAs (miRNAs) are small interfering RNAs that act in post-transcriptional repression. Many studies have indicated that arecoline dysregulates several miRNAs. Recent studies have suggested that arecoline can repress p53, which is necessary to induce miR-22 expression [6, 7]. In addition, c-Myc also directly suppresses miR-22 expression [8]. Furthermore, miR-22 acts as a tumor suppresser in a variety of cancers [9, 10]. However, the role of miR-22 on OSCC remains unknown.

Oncostatin M (OSM) is an IL-6 family inflammatory cytokine which has a number of properties. It is mainly produced in neutrophils, T lymphocytes, macrophages as well as cancer cells [11]. However, the role of OSM in carcinogenesis is still debated. Some reports indicated that OSM inhibits tumor growth and metastasis in melanoma [12], lung cancer [13], etc. Inversely, OSM has been reported to induce tumor growth and metastasis in ovarian cancer [14], breast cancer [15] and osteosarcoma [16]. The function of dysregulated endogenous OSM in cancer cell lines, including in OSCC cell lines, is still unknown.

In present study, we hypothesized that arecoline induces oral carcinogenesis by increasing c-Myc expression, consequently reducing miR-22 levels causing dysregulation of OSM. Thereby, the effects of arecoline on cell viability and cell-cycle progression of OSCC cells were investigated. The corresponding expressions of various target genes including IL-6, STAT3, c-Myc and miR-22 as well as OSM were also determined. In addition, the effects of miR-22 on post-transcriptional repression of OSM as well as miR-22 functions were studied to more elucidate mechanism by which arecoline might influence OSCC development/progression.

## Materials and methods

### Cell line and cell culture

Human OSCC cell lines; ORL-48(T) which is well differentiated SCC cell line that originated from mouth/gum with non-betel quid habit and ORL-136(T) which is well differentiated SCC cell line that originated from tongue with betel quid habit, kindly provided by Prof. Sok Ching Cheong (Cancer Research Initiatives Foundation, Sime Darby Medical Centre Jaya, Malaysia), were cultured in DMEM/F12 (Gibco-Life Technologies, Grand Island, NY, USA) supplemented with 10% fetal bovine serum (FBS) (Gibco-Life Technologies), hydrocortisone (Sigma-Aldrich, Taufkirchen, Germany) and antibiotics (Gibco-Life Technologies) [17]. Human embryonic kidney 293FT cell line (HEK 293FT, Invitrogen, Carlsbad, CA, USA) was maintained in DMEM supplemented with 10% FBS and antibiotics. All of them were maintained in an incubator with an atmosphere at 5% CO_2_ and at 37°C.

### pGL3-Basic vector carrying the c-*myc* promoter

PCR was used to amplify the c-*myc* core promoter from HeLa genomic DNA using the c-Myc promoter primer as shown in Table 1. PCR conditions are described in Supporting information: S1 Table. The 468 bp PCR product was purified using a HiYield^™^ Gel/PCR DNA Fragments Extraction Kit (RBC Bioscience, Taipei, Taiwan) and cloned into pGEM-T vector (Promega, Madison, WI, USA). The constructed plasmid was transformed into *Escherichia coli* (*E*. *coli*) strain DH5α. The product containing c-*myc* core promoter in pGEM-T vector was subcloned into the pGL3-Basic vector, which lacks eukaryotic promoter sequences and contains the firefly luciferase (Promega) as a reporter. The c-*myc* core promoter sequence was confirmed by sequencing analysis.

**Table 1:** Primer sequences.

Primer	Oligonucleotide sequence	Size (bp)
IL-6	F: 5′-CTTCGGTCCAGTTGCCTTCT-3′ R: 5′-TGGAATCTTCTCCTGGGGGT-3′	86 [18]
STAT3	F: 5′-CTGGCCTTTGGTGTTGAAAT-3′ R: 5′-AAGGCACCCACAGAAACAAC-3′	202 [19]
c-Myc	F: 5′-CCACTCGGAAGGACTATCCTGCTG-3′ R: 5′-GCGCTCCAAGACGTTGTGTGTTCG-3′	152
c-Myc promoter	F: 5ʹ-GGTACCTCCTCTCTCGCTAATCTCCGC-3ʹ R: 5′-AAGCTTCGGGAGGGCTGGGCCAGA-3ʹ	468
OSM	F: 5′-CTCGAAAGAGTACCGCGTG-3′ R: 5′-TCAGTTTAGGAACATCCAGGC-3′	119 [20]
miR-22	F: 5ʹ-AGCG GACGCAGTGATTTGCT-3ʹ R: 5ʹ-AACGTATCATCCACCCTGCT-3ʹ	347
miR-22 cloning	F: 5′-GGTACCAGCGGACGCAGTGATTTGCT-3′ R: 5′-GGATCCAACGTATCATCCACCCTGCT-3′	359
OSM 3'UTR WT	F: 5'-CAGTCTAGACATTGATTCAGGGGTCTGATGACAC-3' R: 5'- CGTCTAGAAGGGAATCCAAGCAACCGACAGG-3'	433
OSM 3'UTR Mut	F: 5'-GACCTAACTTTACGGAGGTGTAACAGCG-3′ R: 5’-CGCTGTTACACCTCCGTAAAGTTAGGTC-3′	428
Actin	F: 5′-TCACCCACACTGTGCCCATCTACGA-3ʹ R: 5ʹ-CAGCGGAACCGCTCATTGCCAATGG-3ʹ	294 [21]
GAPDH	F: 5′-TCATCAGCAATGCCTCCTGCA-3ʹ R: 5′-TGGGTGGCAGTGATGGCA-3ʹ	117 [22]

### pIRES-miR-22 vector

The miR-22 fragment containing the stem-loop sequence was amplified from ORL-48(T) cDNA using specific primers as shown in Table 1: Hsa-miR-22-forward and reverse primers (with *Kpn*I and *Bam*HI restriction site, respectively). PCR conditions are described in Supporting information: S1 Table. The fragment was cloned into pGEM-T vector and then subcloned into pIRES2-EGFP vector (Clontech, Palo Alto, CA, USA).

### pGL3-OSM 3ʹUTR (untranslated region) WT and Mut vectors

OSM 3ʹUTR WT were amplified from ORL-48(T) cDNA using specific primers with *Xba*I restriction site (Table 1). The mutant of OSM 3ʹUTR was amplified by PCR-based site-directed mutation using OSM 3′-UTR Mut-forward and reverse primers. PCR conditions are described in Supporting information: S1 Table. Both OSM 3ʹUTR WT and Mut were cloned into pGEM-T vector and then subcloned into *Xba*I-digested pGL3-Control vector (Promega).

### MTT assay for determination of cell cytotoxicity and cell proliferation

To determine cytotoxicity of arecoline, 4 x 10^4^ cells of ORL-48(T) or ORL-136(T) cells were seeded into each well of 96-well tissue culture plates, and maintained in complete medium for 24 hours. The cells were treated with arecoline (Sigma-Aldrich, St. Louis, MO, USA) at 0, 25, 50, 100, 200, 400, 800 and 1,200 μg/ml in triplicate for 24 hours. Cell viability was determined by MTT assay.

To determine the effect of arecoline on cell proliferation, 5 x 10^3^ cells of ORL-48(T) and ORL-136(T) cells in serum-starved DMEM/F12 medium were seeded into each well of 96-well tissue culture plates for 24 hours. The cells were treated with arecoline at 0, 0.025, 0.25, 2.5 and 25 μg/ml in serum-starved DMEM/F12 medium in triplicate for a further 24 hours. Cell proliferation was determined by the MTT assay.

By MTT assay, 10 μl MTT (5 mg/ml) was added to each well. After 4 hours, the medium was removed and the water-insoluble purple formazan particles were dissolved in 100 μl DMSO solution. The absorbance was read at 570 nm with a Microplate Reader (TECAN, Salzburg, Austria).

### Flow cytometry for cell-cycle analysis in arecoline-treated and miR-22-transfected ORL-48(T) cells

In arecoline-treated cells, 10^5^ ORL-48(T) cells were seeded into 6-well tissue culture plates. Cells were synchronized by serum starvation for 24 hours then treated with 0, 0.025 and 25 μg/ml arecoline for 24 hours. For miR-22-treated and untreated cells, 10^5^ ORL-48(T) cells were transfected with mock control and pIRES-miR-22 for 6 hours and then transfected cells were cultured in complete medium (DMEM + 10% FBS) and incubated for 24 hours. In both experiments, cells were detached with 0.25% trypsin solution, washed twice with phosphate-buffered saline (PBS) and fixed with 70% ethanol at 4°C for 24 hours. Propidium iodide (PI) solution containing 1X binding buffer, 20 μg/ml PI (BD Pharmingen, Heidelberg, Germany) and 0.5U RNase A (Sigma) was freshly prepared to stain the cells. After incubation for 20 minutes, cells were analyzed using flow cytometry (Becton Dickinson FACSCanto II, USA). Triplicate independent experiments were performed.

### RT-PCR for determination of IL-6, STAT3, c-Myc, miR-22 and OSM expression

RNA was extracted from cells including arecoline-treated/untreated cells; ORL-48(T) and ORL-136(T) cells and miR-22-transfected/untransfected cells; ORL-48(T) and ORL-136(T) cells using TRIzol^®^ reagent (Invitrogen, Carlsbad, CA). cDNA was synthesized from the extracted RNA with an Oligo (dT) primer using a SuperScript III First-Strand Synthesis System (Invitrogen) according to the manufacturer’s instruction. The cDNA was used as the template to determine the expression of OSM, IL-6, STAT3, c-Myc and miR-22 using RT-PCR. GAPDH (for OSM, IL-6, STAT3 and c-Myc) and β-actin (for miR-22) were use as internal controls. Each reaction consisted of 4 μl of cDNA, 1X SsoAdvanced^™^ Universal SYBR^®^ Green Supermix (Bio-Rad Laboratories, Hercules, CA), 300 nM sense and 300 nM antisense primers. The RT-PCR was performed in the Light Cycler 480 (Roche Applied Science, Indianapolis, IN, USA). Fold change expression was calculated using ΔΔCt and relative to the untreated group. The primer sequences and RT-PCR conditions were shown in Table 1 and Supporting information: S2 Table. Each experiment was performed in triplicate.

### Western blotting for determination of c-Myc and OSM protein

Protein from arecoline-treated and untreated, and miR-22 transfected ORL-48(T) and ORL-136(T) cells was isolated with radioimmunoprecipitation assay (RIPA) buffer (25 mM Tris-HCl pH 7.6, 150 mM NaCl, 1% sodium deoxycholate, 0.1% SDS). OSM, c-Myc and actin proteins were determined by western blot using as primary antibodies rabbit anti-OSM (1:100 dilution; Sigma), mouse anti-c-Myc (1:100 dilution; clone 9E10, Santa Cruz Biotechnology, Santa Cruz, CA, USA) and mouse anti-actin (1: 1,000 dilution; Sigma). The intensity of protein bands was measured using Image J 1.49v software (National Institutes of Health, Bethesda, MD, USA).

### Determination of arecoline targeting c-*myc* promoter activity

500 ng pGL3-c-*myc* promoter (cMYCP) or pGL3-Basic (mock) vector was transfected into 4 x 10^4^ ORL-48(T) cells in 96-well plates for 6 hours and then maintained in DMEM complete medium with/without arecoline treatment for 24 and 48 hours. c-*myc* promoter activity was measured by luciferase assay using Bright-Glo™ system (Promega). The luciferase activity unit in pGL3-cMYCP-transfected cells was normalized with arecoline-treated mock controls and relative to the untreated group. This experiment was performed in triplicate.

### Luciferase assay for determination of miR-22 targeting OSM 3′UTR-WT and OSM 3′UTR-Mut

250 ng pIRES-miR-22 and 100 ng pGL3-OSM 3′UTR WT or Mut vectors were co-transfected into 293FT cells. At 24 and 48 hours post transfection, luciferase activity in co-transfected cells was measured using the Bright-Glo™ system. Luciferase activity of p IRES-miR-22 and pGL3-OSM 3′UTR WT or Mut co-transfected cells was normalized against luciferase activity in cells co-transfected with pIRES2-miR-22- and pGL3-Control vectors, and was relative to the normalized luciferase activity of pIRES2-EGFP and OSM 3′UTR co-transfected cells with pIRES2-EGFP and pGL3-control co-transfected cells. This experiment was performed in triplicate.

### Wound healing assay for determination of cell migration

2 x 10^5^ ORL-48(T) cells were seeded into 24-well tissue culture plates for 24 hours and transfected with pIRES-miR-22 vector using Lipofectamine^®^2000. After transfection for 6 hours, the monolayer was gently and slowly scratched with a 10 μl pipette tip. Wound closure was determined at 0, 24, 48 and 72 hours under a microscope. Extent of wound closure was measured using NIS-Elements Advanced Research Imaging Software version 3.0. The cell migration assay was performed in triplicate in separate wells.

### Statistical analysis

Data are expressed as mean ± SEM (standard error of the mean). *, ** and *** were denoted as significant difference in *P* < 0.05, 0.01 and 0.001, respectively. Paired *t*-test was used for cell-cycle analysis. One-way ANOVA followed by Tukey’s multiple comparison test was used to analyze cell viability and RT-PCR results in arecoline-treated and -untreated cells. Two-way ANOVA was used to analyze the significant level of luciferase activity between and within groups. All statistical analysis was performed using Prism5 software (GraphPad, San Diego, CA, USA).

## Results

### High doses of arecoline were cytotoxic but low doses induced cell proliferation and cell-cycle progression

**Fig 1:**
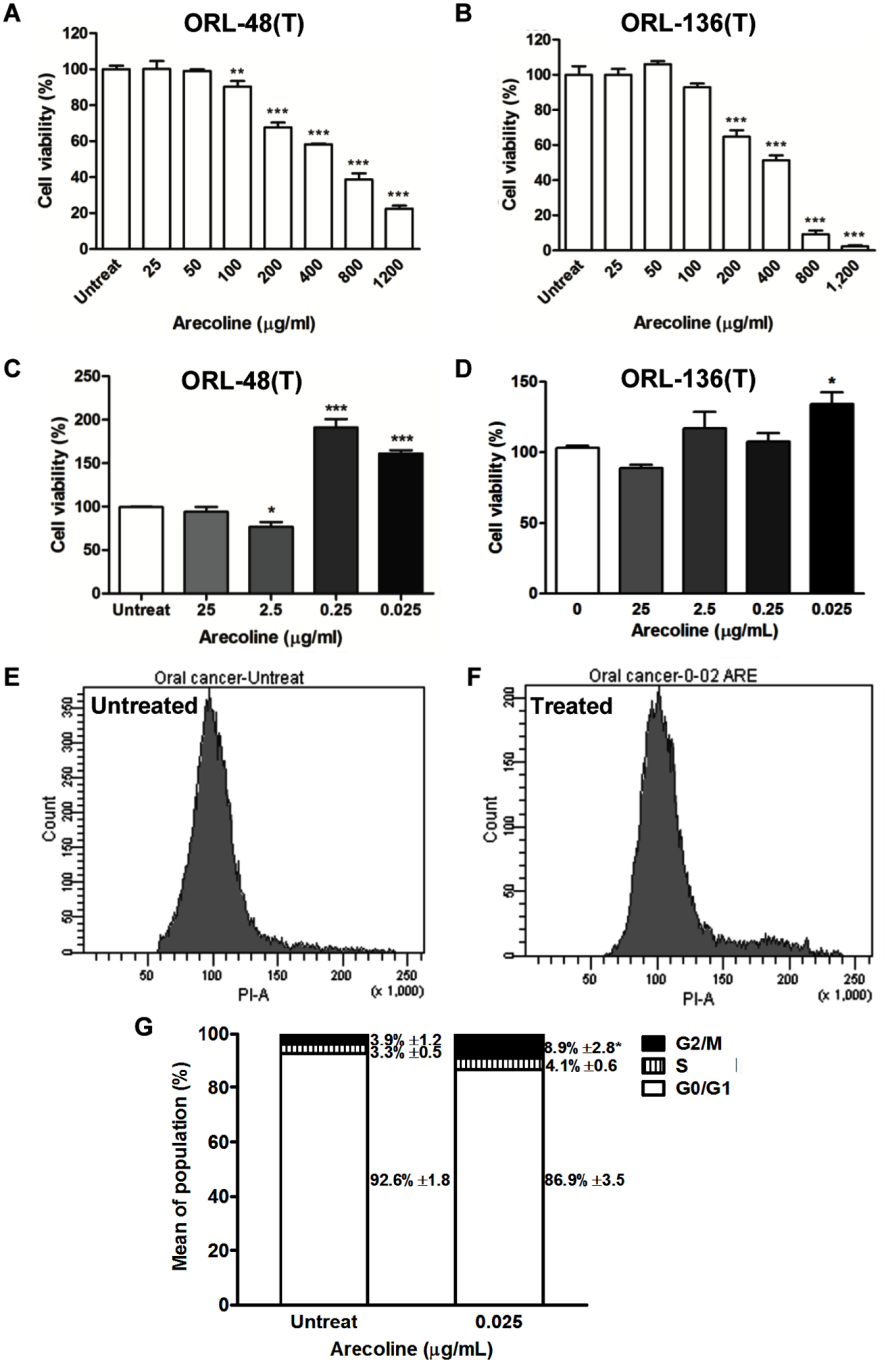
The effects of arecoline on cell viability and cell-cycle progression. Cytotoxicity (A and B) and cell proliferation (C and D) were determined in arecoline-untreated or treated OSCC cell lines at various concentrations for 24 hours using the MTT assay. Statistical significance of the differences of cell viability (%) was analyzed using One-way ANOVA followed by Tukey’s multiple comparison test (*P < 0.05, **P < 0.01 and ***P < 0.001). Cell-cycle phase distribution (E and F) in ORL-48(T) cells treated with 0 and 0.025 μg/ml of arecoline in synchronized condition was analyzed by flow cytometry. The percentages of G0/G1, S and G2/M population (G) of arecoline-treated cells were compared to untreated ORL-48(T) cells as control. Statistical significance of the differences of G2/M population was analyzed using Paired *t*-test (*P < 0.05).

To investigate the cytotoxicity of arecoline, ORL-48(T) and ORL-136(T) cell lines were treated with different concentrations of arecoline for 24 hours. Fig 1A and 1B showed cytotoxic levels of arecoline on ORL-48(T) and ORL-136(T) cells that were higher than 100 and 200 μg/ml, respectively. Arecoline at low concentration increased viability of both ORL-48(T) and ORL-136(T) cells (Fig 1C and 1D). These results demonstrated that arecoline at 0.025 μg/ml increased cell viability of OSCC cell lines, therefore, this concentration was used in further experiments.

The effect of arecoline on cell-cycle progression was confirmed by flow cytometry. Arecoline at 0.025 μg/ml induced significant proliferation of ORL-48(T) cells by increasing the proportion of G2/M phase (8.9 ± 2.8% G2/M cells) when compared to untreated cells (3.9 ± 1.2% G2/M cells) as shown in Fig 1E, 1F and 1G.

### The effects of arecoline on c-*myc* promoter and expression

**Fig 2:**
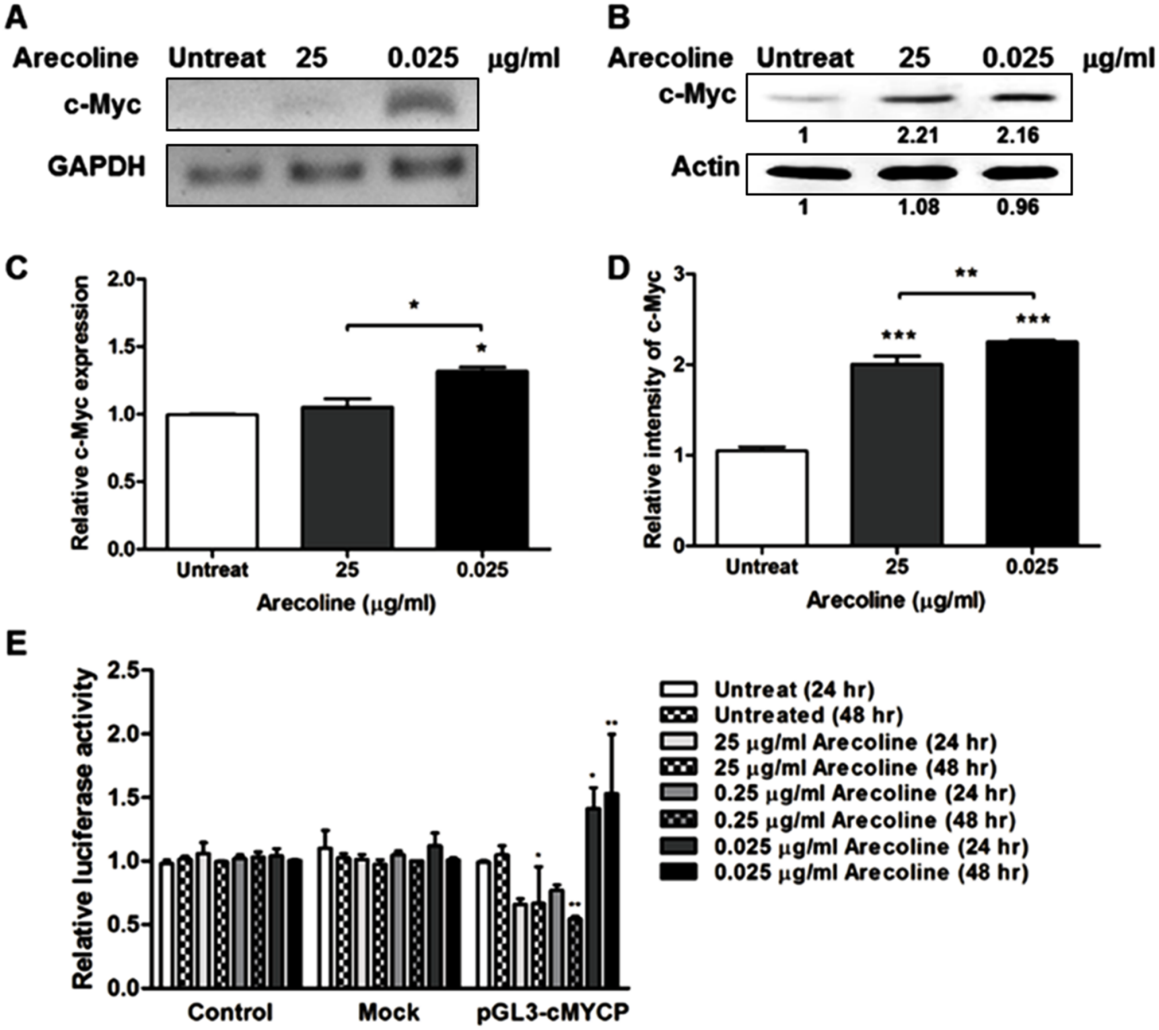
The effects of arecoline on c-Myc expression and transcriptional activity. ORL-48(T) cells treated with 0, 0.025 and 25 μg/ml of arecoline for 24 hours were assayed to determine levels of c-Myc expression in mRNA (RT-PCR) (A) and protein (western blot) (B). Relative c-Myc expression and relative intensity of c-Myc protein band were investigated in RT-PCR and western blot result (C-D). Statistical significance of the differences was analyzed using One-way ANOVA followed by Tukey’s multiple comparison test (*P < 0.05, **P < 0.01 and ***P < 0.001). Mock or pGL3-cMYCP vector-untransfected or transfected ORL-48(T) cells were treated with 0, 0.025, 0.25 and 25 μg/ml of arecoline for 24 and 48 hours (E). The transcriptional activity of the c-*myc* promoter was determined by luciferase activity. Statistical significance of the differences of luciferase activity was analyzed using Two-way ANOVA (*P < 0.05, **P < 0.01 and ***P < 0.001).

c-Myc is a transcriptional activator and repressor of various target genes, contributing to many biological processes especially cell proliferation [5]. c-Myc is a likely target for arecoline. However, the effect of arecoline on c-Myc expression is debated. In order to explore this effect, expression of c-Myc in arecoline-treated ORL-48(T) cells was assayed using real time polymerase chain reaction (RT-PCR) and western blot. In the arecoline-treated cells, c-Myc expression was increased at both the mRNA and protein levels (Fig 2A and 2B). The level of c-Myc mRNA and protein in cells treated with 0.025 μg/ml arecoline was significantly higher than in the other treatments (Fig 2C and 2D). This indicates that arecoline can upregulate c-Myc expression.

To confirm this, a pGL3-Basic vector containing the c-*myc* core promoter (P1 and P2 regions) was constructed. ORL-48(T) cells were transfected with this vector. From 24 hours post-transfection, the cells were incubated with various concentrations of arecoline for 24 and 48 hours. Arecoline was shown to induce transcriptional activity of the c-*myc* promoter as shown in Fig 2E. At 0.025 μg/ml arecoline treatment, relative luciferase activity was significant higher than in untreated cells for 24 and 48 hours. This demonstrates that the low concentration (0.025 μg/ml) of arecoline could induce transcriptional activity of c-*myc* promoter, resulting in c-Myc upregulation. However, at higher concentrations of arecoline, transcriptional activity of the c-*myc* promoter was decreased.

### Arecoline can induce IL-6/STAT3 upstream of c-Myc

**Fig 3:**
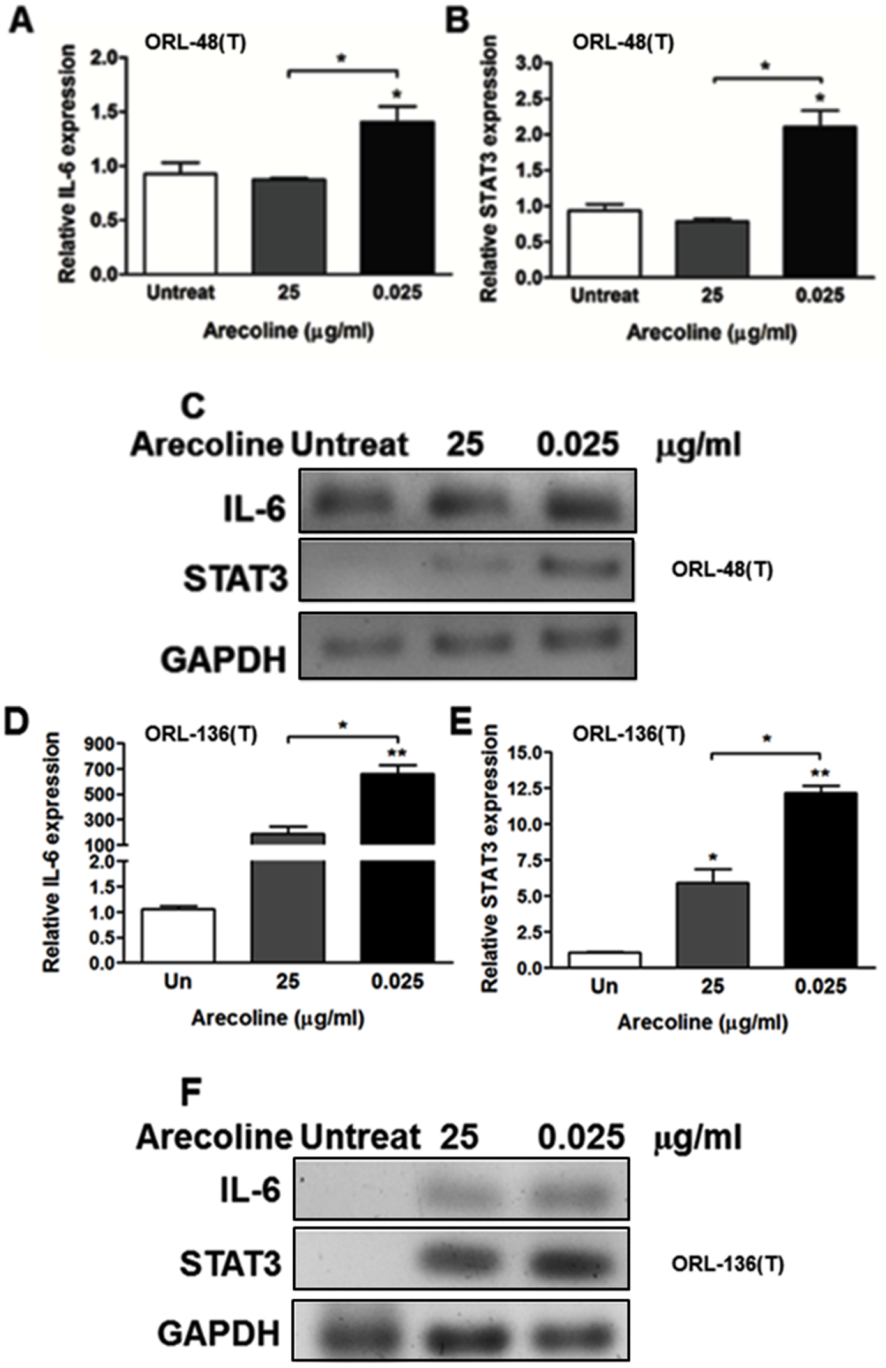
Effect of arecoline on IL-6 and STAT3 in ORL-48(T) and ORL-136(T) cells. ORL-48(T) and ORL-136(T) cells were treated with 0, 0.025 and 25 μg/ml arecoline for 24 hours. Expression of IL-6 (A and D) and STAT3 (B and E) were investigated by RT-PCR and their amplicons were visualized by 2% agarose gel electrophoresis (C and F). Statistical significance of the differences of relative expression was analyzed using One-way ANOVA followed by Tukey’s multiple comparison test (*P < 0.05 and **P < 0.01).

IL-6/STAT3 signaling cascade induces many downstream targets and its dysregulation could contribute to initiation, promotion, and progression of tumor-associated inflammation [23]. c-Myc is a well-known target of IL-6/STAT3 [5]. Previous studies revealed that areca nut extract could induce IL-6 production, while arecoline decreased IL-6 levels [24]. In our finding, a low concentration of arecoline could induce c-Myc transcriptional activity. To more clarify the possible involvement of IL-6 and STAT3, therefore, ORL-48(T) and ORL-136(T) cells were treated with arecoline and investigated for IL-6 and STAT3 expression using RT-PCR. Expression of both IL-6 and STAT3 in ORL-48(T) seemed to decrease in cells treated with 25 μg/ml of arecoline, whereas it was significantly highest in cells treated with 0.025 μg/ml arecoline in both ORL-48(T) and ORL-136(T) cells (Fig 3). This result indicated that different arecoline concentrations and cell types may impact expression of its target genes. At 0.025 μg/ml arecoline could induce IL-6/STAT3 expression, possibly causing upregulation of the downstream target, c-Myc.

#### The effect of arecoline on miR-22 expression in OSCC cell lines

**Fig 4:**
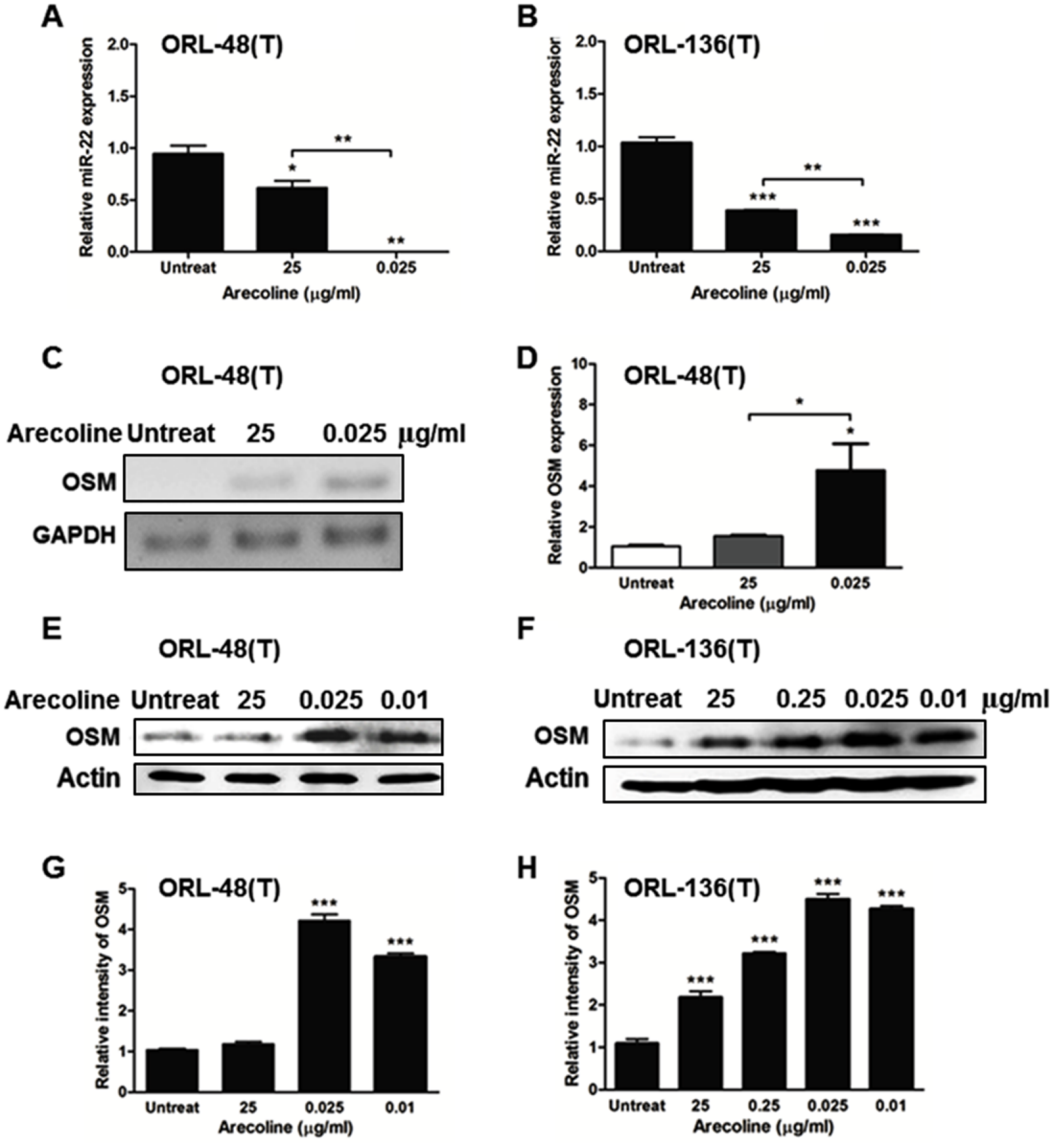
The effects of arecoline on miR-22 and OSM expression. Both ORL-48(T) and ORL-136(T) cells were exposed to 0, 0.025 and 25 μg/ml of arecoline for 24 hours. Pri-miR-22 expression was detected by RT-PCR. β-actin was used as internal controls (A-B). ORL-48(T) cells were treated with various concentrations of arecoline for 24 hours; then OSM mRNA expression in these cells was analyzed using RT-PCR (C-D). OSM protein levels in ORL-48(T) (E) and ORL-136(T) (F) cells were examined by western blot and relative intensities were analyzed by ImageJ 1.49v software (G-H). Statistical significance of the differences of relative expression was analyzed using One-way ANOVA followed by Tukey’s multiple comparison test (*P < 0.05, **P < 0.01 and ***P < 0.001).

Some studies have demonstrated that arecoline had a comprehensive effect on cellular gene expression, including expression of miRNA [25]. The role of arecoline in miRNA expression has received little investigation. A previous study have suggested that arecoline repressed expression of p53 [26], a protein that directly upregulates miR-22 [10]. Inversely, c-Myc directly inhibited miR-22 expression [8]. In addition, the role of miR-22 in OSCC has remained unclear. Therefore, to more elucidate the role of arecoline in epigenetic alteration especially tumor suppressing miR-22 in OSCC, ORL-48(T) and ORL-136(T) cells were treated with arecoline at 0, 0.025 and 25 μg/ml and the level of pri-miR-22 was examined by RT-PCR. In cells treated with 0.025 and 25 μg/ml of arecoline, miR-22 expression was significantly reduced (Fig 4A and 4B), in contrast to untreated cells. This result indicated that miR-22 could be suppressed by arecoline in OSCC.

### OSM was a putative target of miR-22 and upregulated by arecoline

From *in silico* results, OSM is predicted as a target of miR-22 according to algorithms in TargetScanHuman Release 6.2 [27] and miRNA.org [28]. Interestingly, OSM promoted tumor growth and progression in several cancers [11]. OSM induced IL-6 and STAT3, with subsequent effects on many signaling cascades [29] and also induced c-Myc expression [30]. Moreover, dysregulation of c-Myc switched OSM function from cancer suppression to cancer promotion because OSM-induced senescence was inhibited by c-Myc [11]. Therefore, we aimed to investigate roles of arecoline and miR-22 on OSM expression in OSCC. We determined OSM mRNA and protein in ORL-48(T) and ORL-136(T) cell lines treated various concentrations of arecoline for 24 hours. OSM mRNA was significantly higher in cells treated with 0.025 μg/ml arecoline than in untreated cells (Fig 4C and 4D). Concordantly, OSM protein levels in ORL-48(T) and ORL-136(T) cells treated with arecoline were increased as shown in Fig 4E, 4F, 4G and 4H. This result firstly demonstrates that OSM expression is induced by arecoline and OSM is negatively correlated with miR-22.

### OSM is a target of miR-22

**Fig 5:**
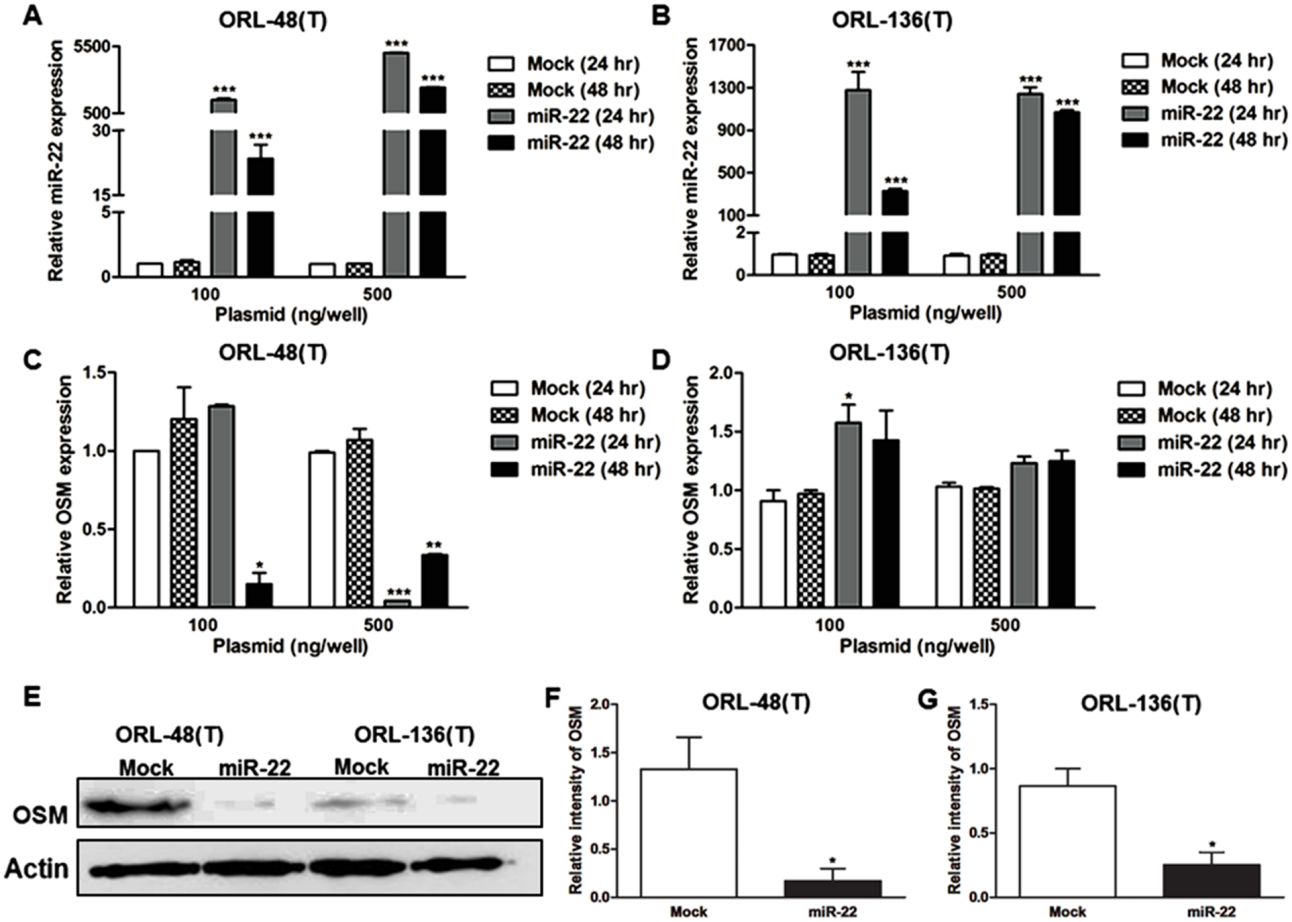
Relative expression levels of miR-22 and OSM. 100 and 500 ng/well of mock control (pIRES2-EGFP vector) and pIRES-miR-22 vectors were transfected into ORL-48(T) and ORL-136(T) cells. At 24 and 48 hours post-transfection, pri-miR-22 (A-B) and OSM (C-D) expression was determined by RT-PCR. Protein levels of OSM (E) were determined in ORL-48(T) and ORL-136(T) cells at 48 hours after transfection with 100 ng of mock control and pIRES-miR-22 vectors. Relative intensity of OSM protein band (F-G) was calculated using ImageJ 1.49v software. Statistical significance of the differences was analyzed using One-way ANOVA followed by Tukey’s multiple comparison test (*P < 0.05, **P < 0.01 and ***P < 0.001).

To further explore the negative correlation between miR-22 and OSM, two concentrations of pIRES-miR-22 vector were transfected into ORL-48(T) and ORL-136(T) cells. Fig 5A and 5B show miR-22 expression. Both mRNA and protein levels of OSM in ORL-48(T) cells transfected with pIRES-miR-22 were reduced when compared with controls (Fig 5C, 5E and 5F) whereas only OSM protein in pIRES-miR-22-transfected ORL-136(T) cells was reduced (Fig 5E and 5G). These results demonstrate that miR-22 reduces OSM expression and suggest that OSM may be a direct target of miR-22.

### MiR-22 directly targets OSM

**Fig 6:**
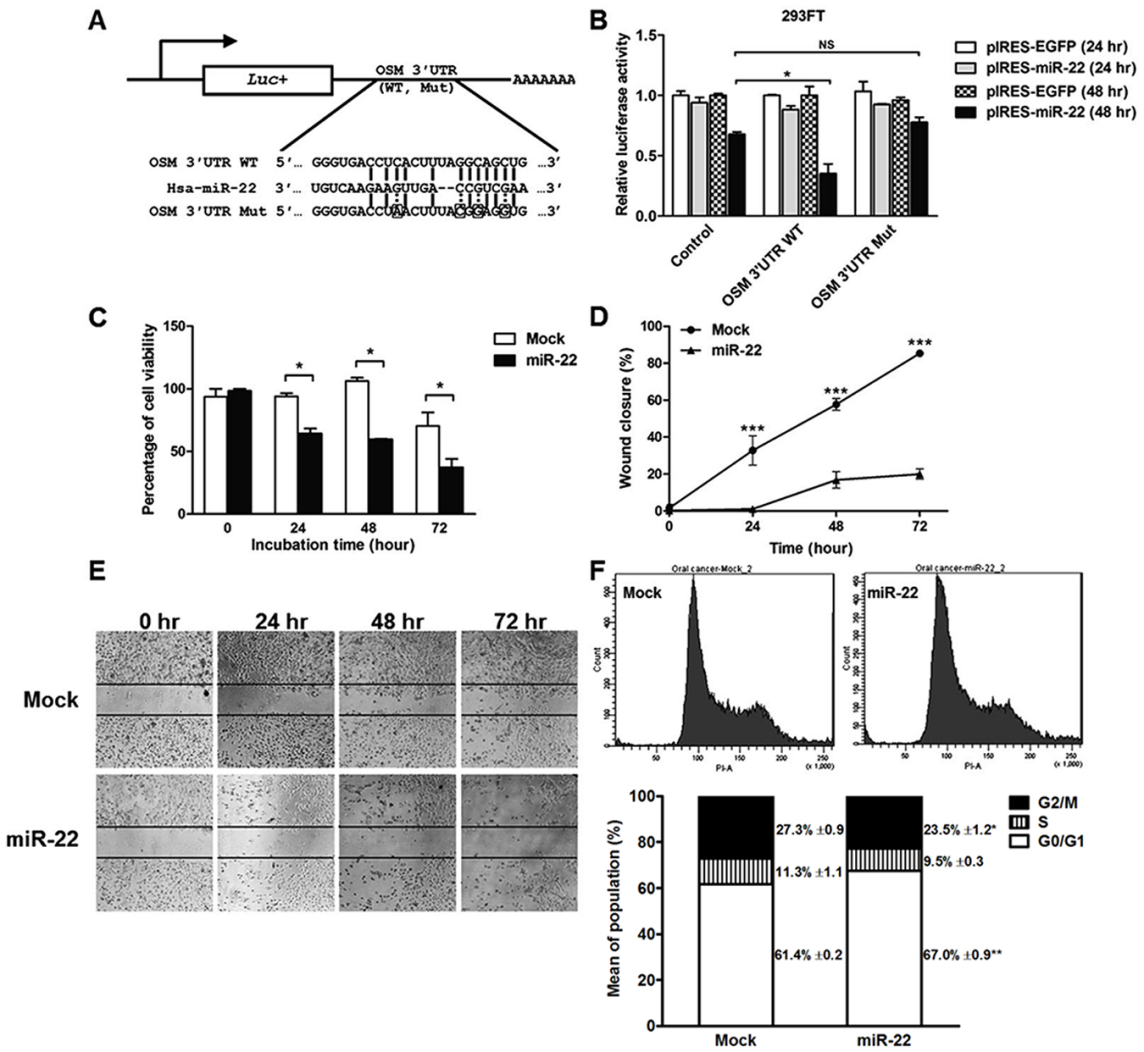
MiR-22 targets OSM and miR-22 functions in cell proliferation, migration and cell-cycle assay. The construct of the miR-22 targets sequence within the OSM 3′UTR WT and Mut in pGL3-Control vector. The luciferase gene was linked to the 3′UTR WT and Mut of OSM. 293FT cells were co-transfected with 250 ng pIRES-miR-22 and 100 ng pGL3-OSM 3′UTR WT or Mut vectors (A). The normalized luciferase activity in pIRES-miR-22 and pGL3-OSM 3′UTR WT or Mut co-transfected cells was relative to normalized luciferase activity of pIRES2-EGFP and OSM 3′UTR WT or Mut co-transfected cells (B). A green fluorescence expression vector (pEGFP-N3) was transfected for monitoring transfection efficiency. Statistical significance of the differences of luciferase activity was analyzed using Two-way ANOVA (*P < 0.05). Cell proliferation and migration in pIRES-miR-22-transfected ORL-48(T) cells were measured by a hemocytometer and wound healing assay at different incubation time points (C-E). The photograph was taken under 4X objective lens NIS-Elements Advanced Research Imaging Software version 3.0. Statistical significance of the differences of cell viability and wound closure was analyzed using Student's *t*-test (*P < 0.05 and ***P < 0.001). Cell-cycle assay in miR-22 or mock-transfected ORL-48(T) for 48 hours post-transfection was performed by flow cytometry (F). Statistical significance of the differences of G2/M and G0/G1 population was analyzed using Paired *t*-test (*P < 0.05 and (**P < 0.01, respectively).

To further confirm that OSM is a direct target of miR-22, we tested whether miR-22 could suppress the 3′UTR of OSM. The OSM 3′UTR wild type (WT) or mutant (Mut) was cloned on downstream of the firefly luciferase gene in the pGL3-Control vector (Fig 6A). pGL3-OSM 3′UTR WT or Mut plasmid was co-transiently transfected into 293FT cells along with pIRES-miR-22 vectors or pIRES2-EGFR vector (negative control). Luciferase activity in pIRES-miR-22 and pGL3-OSM 3′UTR WT co-transfected cells was significantly decreased at 48 hours post-transfection but not in pGL3-OSM 3′UTR Mut compared to negative controls as shown in Fig 6B. This result revealed that OSM is the direct target of miR-22.

### MiR-22 suppresses cell proliferation, migration and cell-cycle progression of OSCC cells

MiR-22 functions in OSCC cell line were determined in pIRES-miR-22-transfected ORL-48(T) cells. At 0, 24, 48 and 72 hours post-transfection, cell proliferation and migration were measured by a hemocytometer and wound healing assays, respectively. As expected, viability of cells with overexpressed miR-22 was lower than mock controls (pIRES2-EGFP-transfected cells) (Fig 6C). Moreover, migration of miR-22-transfected cells was suppressed, resulting in a lower extent of wound closure as shown in Fig 6D and 6E. Furthermore, cell population in G2/M phase of miR-22-transfected cells was significantly reduced when compared with mock controls (Fig 6F). This result has inversely correlated with arecoline-induced cell-cycle progression in G2/M phase (Fig 1G). Importantly, miR-22 that could be reduced by arecoline, acts as a tumor suppresser that suppresses cell proliferation, migration and cell-cycle progression in OSCC cells.

## Discussion

This study investigated the effects of various concentrations of arecoline on viability and proliferation of OSCC cells. We found that low concentration of arecoline induced proliferation and cell-cycle progression at the G2/M phase, whereas high concentration induced cell death. The effects of arecoline, therefore depend on its concentration and cell types [26, 31].

A report has indicated that a high dose of arecoline caused cell death in gingival keratinocytes (0.8–1.2 mM arecoline; ~ 188.8–283.3 μg/ml) and in oral KB carcinoma cells (0.4–1.2 mM arecoline; ~ 94.4–283.3 μg/ml) [24]. In agreement with our work, arecoline concentration lower than 0.8 μg/ml enhanced cell growth of oral fibroblasts, epidermal cells of the mouth and OSCC cell lines, whereas arecoline at higher concentrations (25–400 μg/ml) was cytotoxic [32].

Arecoline likely regulates c-Myc, which is well known to be a key driver of cell proliferation contributing to tumorigenesis [6, 33]. In this study, we found that arecoline can induce c-*myc* promoter transcriptional activity leading to high level expression of c-Myc protein. A recent study has suggested that arecoline can reduce IL-6 and STAT3 in a human hepatoma cell line at concentrations of 0, 3, 30 and 100 μg/ml [31]: in contrast, we found that arecoline treatment at 0.025 μg/ml could upregulate IL-6 and STAT3 mRNA expression in ORL-48(T) cells. These effects may also be linked to c-Myc upregulation. However, the exact mechanism of arecoline-induced IL-6/STAT3/c-Myc expression remains to be explored.

MiR-22 represses transcription of many gene targets, thereby having an important role in tumorigenesis [4], and is often downregulated in various cancers including lung cancer, colorectal cancer and breast cancer [9, 34, 35]. It also represses translation processes of many oncogenes such as SIRT1, Sp1, and CDK6, which are involved in cancer progression [4]. Interestingly, p53 tumor suppresser gene is a direct transcriptional factor for miR-22 [26]. Inversely, c-Myc has a direct inhibitory effect on expression of miR-22 [8]. Simultaneously, the role of this miR-22 in OSCC remains unclear. Therefore, we also interest the role of miR-22 in OSCC. In our findings, miR-22 is downregulated in arecoline-treated OSCC cells. These results reveal for the first time that arecoline downregulates miR-22. It is possible that arecoline downregulates miR-22 via arecoline-induced c-Myc upregulation. The predictive mRNA target of miR-22 was analyzed by *in silico* method and OSM was shown to be a very attractive target of this miRNA. In the present study, we found an inverse correlation of miR-22 and OSM expression in arecoline-treated cells and miR-22-overexpressing OSCC cells. Expectedly, the OSM 3′UTR WT is directly target for miR-22 but not OSM 3′UTR Mut. Part of the seed sequence of miR-22 could be recognized in the OSM 3′UTR sequence. Therefore, this is the first report suggests that arecoline can upregulate OSM expression by suppressing miR-22. In addition, miR-22 can suppress the proliferation, migration and cell-cycle progression in OSCC cell lines. Corresponding with a previous report, miR-22 suppressed cell proliferation and motility of tongue SCC cells [36]. Reduced OSM by miR-22 overexpressing OSCC cells may be involved in cell proliferation and migration [37]. Moreover, OSM has been found to upregulated c-Myc expression in breast cancer cell lines, led to induction of the epithelial-mesenchymal transition (EMT), resulting in tumor progression [38]. So it seems that arecoline-induced c-Myc dyregulation may impact on miR-22 repression targeting OSM that possible promote the proliferation and cell-cycle progression in OSCC. However, the role of OSM in OSCC need to more clarified. From the overall results may suggest that arecoline inhibited a tumor suppresser effect of miR-22 targeting OSM, subsequently promoting cell proliferation and cell-cycle progression in OSCC.

## Supporting information

S1 Table PCR conditions. (DOC)

S2 Table Real-time PCR conditions. (DOC)

## Abbreviations

293FT: Human embryonic kidney 293FT cell line
EMT: Epithelial-mesenchymal transition
FBS: Fetal bovine serum
miR: microRNA
MTT: 3-[4,5-dimethylthiazol-2-yl]-2,5-diphenyl tetrazolium bromide
Mut: Mutant
OSCC: Oral squamous cell carcinoma
OSM: Oncostatin M
PBS: Phosphate-buffered saline
RT-PCR: Real time polymerase chain reaction
UTR: Untranslated region
WT: Wild type

## References

[1] Lee CH, Ko AMS, Warnakulasuriya S, Yin BL, Zain RB, Ibrahim SO, et al Intercountry prevalences and practices of betel-quid use in south, southeast and eastern asia regions and associated oral preneoplastic disorders: An international collaborative study by asian betel-quid consortium of south and east Asia. Int J Cancer. 2011;129(7):1741–51. https://doi.org/10.1002/ijc.25809 . 21128235 10.1002/ijc.25809

[2] Lee PH, Chang MC, Chang WH, Wang TM, Wang YJ, Hahn LJ, et al Prolonged exposure to arecoline arrested human KB epithelial cell growth: regulatory mechanisms of cell cycle and apoptosis. Toxicology. 2006;220(2):81–9. https://doi.org/10.1016/j.tox.2005.07.026 . 16413651 10.1016/j.tox.2005.07.026

[3] Chandak RM, Chandak MG, Rawlani SM. Current concepts about areca nut chewing. J Contemp Dent. 2013;3(2):78–81. https://doi.org/10.5005/jp-journals-10031-1041.

[4] Xu D, Takeshita F, Hino Y, Fukunaga S, Kudo Y, Tamaki A, et al miR-22 represses cancer progression by inducing cellular senescence. J Cell Biol. 2011;193(2):409–24. https://doi.org/10.1083/jcb.201010100 . 21502362 10.1083/jcb.201010100 PMC3080260

[5] Li N, Grivennikov SI, Karin M. The unholy trinity: inflammation, cytokines, and STAT3 shape the cancer microenvironment. Cancer Cell. 2011;19(4):429–31. https://doi.org/10.1016/j.ccr.2011.03.018 . 21481782 10.1016/j.ccr.2011.03.018 PMC3111086

[6] Tsai YS, Lin CS, Chiang SL, Lee CH, Lee KW, Ko YC. Areca nut induces miR-23a and inhibits repair of DNA double-strand breaks by targeting FANCG. Toxicol Sci. 2011;123(2):480–90. https://doi.org/10.1093/toxsci/kfr182 . 21750350 10.1093/toxsci/kfr182

[7] Tanaka M, Miyahima A. Onconstatin M, a multifunctional cytokine. Rev Physiol Biochem Pharmacol. 2003;149:39–52. https://doi.org/10.1007/s10254-003-0013-1 . 12811586 10.1007/s10254-003-0013-1

[8] Kong LM, Liao CG, Zhang Y, Xu J, Li Y, Huang W, et al A regulatory loop involving miR-22, Sp1, and c-Myc modulates CD147 expression in breast cancer invasion and metastasis. Cancer Res. 2014;74(14):3764–78. https://doi.org/10.1158/0008-5472 . 24906624 10.1158/0008-5472.CAN-13-3555

[9] Ling B, Wang GX, Long G, Qiu JH, Hu ZL. Tumor suppressor miR-22 suppresses lung cancer cell progression through post-transcriptional regulation of ErbB3. J Cancer Res Clin Oncol 2012;138(8):1355–61. https://doi.org/10.1007/s00432-012-1194-2 . 22484852 10.1007/s00432-012-1194-2 PMC11824200

[10] Tsuchiya N, Izumiya M, Ogata-Kawata H, Okamoto K, Fujiwara Y, Nakai M, et al Tumor suppressor miR-22 determines p53-dependent cellular fate through post-transcriptional regulation of p21. Cancer Res. 2011;71(13):4628–39. https://doi.org/10.1158/0008-5472 . 21565979 10.1158/0008-5472.CAN-10-2475 PMC7425979

[11] Junk DJ, Bryson BL, Jackson MW. HiJAK’d signaling; the STAT3 paradox in senescence and cancer progression. Cancers. 2014;6(2):741–55. https://doi.org/10.3390/cancers6020741 . 24675570 10.3390/cancers6020741 PMC4074801

[12] Lacreusette A, Lartigue A, Nguyen JM, Barbieux I, Pandolfino MC, Paris F, et al Relationship between responsiveness of cancer cells to Oncostatin M and/or IL-6 and survival of stage III melanoma patients treated with tumour-infiltrating lymphocytes. J Pathol. 2008;216(4):451–9. https://doi.org/10.1002/path.2416 . 18798220 10.1002/path.2416

[13] Ouyang L, Shen Ly, Li T, Liu J. Inhibition effect of oncostatin M on metastatic human lung cancer cells 95-D in vitro and on murine melanoma cells B16BL6 in vivo. Biomed Res. 2006;27(4):197–202. https://doi.org/10.2220/biomedres.27.197 . 16971773 10.2220/biomedres.27.197

[14] Li Q, Zhu J, Sun F, Liu L, Liu X, Yue Y. Oncostatin M promotes proliferation of ovarian cancer cells through signal transducer and activator of transcription 3. Int J Mol Med. 2011;28(1):101 https://doi.org/10.3892/ijmm.2011.647 . 21399864 10.3892/ijmm.2011.647

[15] Queen MM, Ryan RE, Holzer RG, Keller-Peck CR, Jorcyk CL. Breast cancer cells stimulate neutrophils to produce oncostatin M: potential implications for tumor progression. Cancer Res. 2005;65(19):8896–904. https://doi.org/10.1158/0008-5472 . 16204061 10.1158/0008-5472.CAN-05-1734

[16] Fossey SL, Bear MD, Kisseberth WC, Pennell M, London CA. Oncostatin M promotes STAT3 activation, VEGF production, and invasion in osteosarcoma cell lines. BMC Cancer. 2011;11(1):125 https://doi.org/10.1186/1471-2407-11-125 21481226 10.1186/1471-2407-11-125 PMC3079692

[17] Hamid S, Lim KP, Zain RB, Ismail SM, Lau SH, Mustafa WMW, et al Establishment and characterization of Asian oral cancer cell lines as in vitro models to study a disease prevalent in Asia. Int J Mol Med. 2007;19(3):453–60. . 17273794

[18] Bumrungthai S, Ekalaksananan T, Evans MF, Chopjitt P, Tangsiriwatthana T, Patarapadungkit N, et al Up-regulation of miR-21 is associated with cervicitis and human papillomavirus infection in cervical tissues. PloS One. 2015;10(5):e0127109 https://doi.org/10.1371/journal.pone.0127109 . 26010154 10.1371/journal.pone.0127109 PMC4444121

[19] Chopjitt P, Pientong C, Bumrungthai S, Kongyingyoes B, Ekalaksananan T. Activities of E6 protein of human papillomavirus 16 Asian variant on miR-21 up-regulation and expression of human immune response genes. Asian Pac J Cancer Prev. 2015;16(9):3961–8. . 25987069 10.7314/apjcp.2015.16.9.3961

[20] West NR, Murphy LC, Watson PH. Oncostatin M suppresses oestrogen receptor-α expression and is associated with poor outcome in human breast cancer. Endocr Relat Cancer. 2012;19(2):181–95. https://doi.org/10.1530/ERC-11-0326 . 22267707 10.1530/ERC-11-0326

[21] Konttinen YT, Li TF, Mandelin J, Ainola M, Lassus J, Virtanen I, et al Hyaluronan synthases, hyaluronan, and its CD44 receptor in tissue around loosened total hip prostheses. J Pathol. 2001;194(3):384–90. https://doi.org/10.1002/1096-9896(200107)194:3<384::AID-PATH896>3.0.CO;2-8 . 11439372 10.1002/1096-9896(200107)194:3<384::AID-PATH896>3.0.CO;2-8

[22] Namwat N, Amimanan P, Loilome W, Jearanaikoon P, Sripa B, Bhudhisawasdi V, et al Characterization of 5-fluorouracil-resistant cholangiocarcinoma cell lines. Chemotherapy. 2008;54(5):343–51. https://doi.org/10.1159/000151541 . 18714155 10.1159/000151541

[23] Rawlings JS, Rosler KM, Harrison DA. The JAK/STAT signaling pathway. J Cell Sci. 2004;117(8):1281–3. https://doi.org/10.1242/jcs.00963. 15020666 10.1242/jcs.00963

[24] Jeng JH, Wang YJ, Chiang BL, Lee PH, Chan CP, Ho YS, et al Roles of keratinocyte inflammation in oral cancer: regulating the prostaglandin E2, interleukin-6 and TNF-α production of oral epithelial cells by areca nut extract and arecoline. Carcinogenesis. 2003;24(8):1301–15. https://doi.org/10.1093/carcin/bgg083 . 12807728 10.1093/carcin/bgg083

[25] Chiang SL, Jiang SS, Wang YJ, Chiang HC, Chen PH, Tu HP, et al Characterization of arecoline-induced effects on cytotoxicity in normal human gingival fibroblasts by global gene expression profiling. Toxicol Sci. 2007;100(1):66–74. https://doi.org/10.1093/toxsci/kfm201. . 17682004 10.1093/toxsci/kfm201

[26] Tsai YS, Lee KW, Huang JL, Liu YS, Juo SHH, Kuo WR, et al Arecoline, a major alkaloid of areca nut, inhibits p53, represses DNA repair, and triggers DNA damage response in human epithelial cells. Toxicology. 2008;249(2):230–7. https://doi.org/10.1016/j.tox.2008.05.007 . 18585839 10.1016/j.tox.2008.05.007

[27] Lewis BP, Burge CB, Bartel DP. Conserved seed pairing, often flanked by adenosines, indicates that thousands of human genes are microRNA targets. Cell. 2005;120(1):15–20. https://doi.org/10.1016/j.cell.2004.12.035 . 15652477 10.1016/j.cell.2004.12.035

[28] Betel D, Wilson M, Gabow A, Marks DS, Sander C. The microRNA. org resource: targets and expression. Nucleic Acids Res. 2008;36(suppl 1):D149–D53. https://doi.org/10.1093/nar/gkm995 . 18158296 10.1093/nar/gkm995 PMC2238905

[29] Van Wagoner NJ, Choi C, Repovic P, Benveniste EN. Oncostatin M regulation of interleukin-6 expression in astrocytes. J Neurochem. 2000;75(2):563–75. https://doi.org/10.1046/j.1471-4159.2000.0750563.x . 10899931 10.1046/j.1471-4159.2000.0750563.x

[30] Dvorak K, Dvorak B. Role of interleukin-6 in Barrett’s esophagus pathogenesis. World J Gastroenterol. 2013;19(15):2307–12. https://doi.org/10.3748/wjg.v19.i15.2307 . 23613623 10.3748/wjg.v19.i15.2307 PMC3631981

[31] Cheng H-L, Su S-J, Huang L-W, Hsieh B-S, Hu Y-C, Hung T-C, et al Arecoline induces HA22T/VGH hepatoma cells to undergo anoikis-involvement of STAT3 and RhoA activation. Mol Cancer. 2010;9(1):1 https://doi.org/10.1186/1476-4598-9-126 . 20507639 10.1186/1476-4598-9-126 PMC2895595

[32] Yang YY, Koh LW, Tsai JH, Tsai CH, Wong EFC, Lin SJ, et al Involvement of viral and chemical factors with oral cancer in Taiwan. Jpn J Clin Oncol. 2004;34(4):176–83. https://doi.org/10.1093/jjco/hyh037 . 15121752 10.1093/jjco/hyh037

[33] Grandori C, Cowley SM, James LP, Eisenman RN. The Myc/Max/Mad network and the transcriptional control of cell behavior. Annu Rev Cell Dev Biol. 2000;16(1):653–99. https://doi.org/10.1146/annurev.cellbio.16.1.653 . 11031250 10.1146/annurev.cellbio.16.1.653

[34] Zhang G, Xia S, Tian H, Liu Z, Zhou T. Clinical significance of miR-22 expression in patients with colorectal cancer. Med Oncol. 2012;29(5):3108–12. https://doi.org/10.1007/s12032-012-0233-9 . 22492279 10.1007/s12032-012-0233-9

[35] Xiong J, Yu D, Wei N, Fu H, Cai T, Huang Y, et al An estrogen receptor α suppressor, microRNA-22, is downregulated in estrogen receptor α‐positive human breast cancer cell lines and clinical samples. FEBS J. 2010;277(7):1684–94. https://doi.org/10.1111/j.1742-4658.2010.07594.x . 20180843 10.1111/j.1742-4658.2010.07594.x

[36] Qiu K, Huang Z, Huang Z, He Z, You S. miR-22 regulates cell invasion, migration and proliferation in vitro through inhibiting CD147 expression in tongue squamous cell carcinoma. Arch Oral Biol. 2016;66:92–7. https://doi.org/10.1016/j.archoralbio.2016.02.013 26943814 10.1016/j.archoralbio.2016.02.013

[37] Kan CE, Cipriano R, Jackson MW. c-MYC functions as a molecular switch to alter the response of human mammary epithelial cells to oncostatin M. Cancer Res. 2011;71(22):6930–9. https://doi.org/10.1158/0008-5472.CAN-10-3860 . 21975934 10.1158/0008-5472.CAN-10-3860 PMC4116142

[38] Guo L, Chen C, Shi M, Wang F, Chen X, Diao D, et al Stat3-coordinated Lin-28–let-7–HMGA2 and miR-200–ZEB1 circuits initiate and maintain oncostatin M-driven epithelial–mesenchymal transition. Oncogene. 2013;32(45):5272–82. https://doi.org/10.1038/onc.2012.573 . 23318420 10.1038/onc.2012.573

