# Supplementary material for: Effects of arecoline on proliferation of oral squamous cell carcinoma cells by dysregulating c-Myc and miR-22, directly targeting oncostatin M
Source: PLoS One. 2018 Jan 31;13(1):e0192009. doi: 10.1371/journal.pone.0192009 (PMC5791990; doi:10.1371/journal.pone.0192009)
Supplement: S1 Table — (DOC) [file pone.0192009.s001.doc]

**S1 Table. PCR conditions.**

| **Primer** | **PCR condition** |
| --- | --- |
| c-Myc promoter | Three min at 95^o^C followed by 40 cycles of 95 ^o^C for 1 min, 60^o^C for 1 min, and 72 ^o^C for 1 min. |
| miR-22 cloning | Three min at 95^o^C followed by 40 cycles of 95 ^o^C for 1 min, 55^o^C for 1 min, and 72 ^o^C for 1 min. |
| OSM 3'UTR WT | Three min at 95^o^C followed by 40 cycles of 95 ^o^C for 1 min, 55^o^C for 1 min, and 72 ^o^C for 1 min. |
| OSM 3'UTR Mut | Three min at 95^o^C followed by 40 cycles of 95 ^o^C for 1 min, 55^o^C for 1 min, and 72 ^o^C for 1 min. |
