# Supplementary material for: Effects of arecoline on proliferation of oral squamous cell carcinoma cells by dysregulating c-Myc and miR-22, directly targeting oncostatin M
Source: PLoS One. 2018 Jan 31;13(1):e0192009. doi: 10.1371/journal.pone.0192009 (PMC5791990; doi:10.1371/journal.pone.0192009)
Supplement: S2 Table — (DOC) [file pone.0192009.s002.doc]

**S2 Table. Real-time PCR conditions.**

| **Primer** | **Real-time PCR condition** |
| --- | --- |
| IL-6 | Two min at 95^o^C followed by 45 cycles of 95 ^o^C for 15 min, 55^o^C for 30 sec, and 72^o^C for 30 sec. |
| STAT3 | Two min at 95^o^C followed by 45 cycles of 95 ^o^C for 15 min, 55^o^C for 30 sec, and 72^o^C for 30 sec. |
| c-Myc | Two min at 95^o^C followed by 45 cycles of 95 ^o^C for 15 min, 55^o^C for 30 sec, and 72^o^C for 30 sec. |
| OSM | Two min at 95^o^C followed by 45 cycles of 95 ^o^C for 15 min, 55^o^C for 30 sec, and 72^o^C for 30 sec. |
| miR-22 | Two min at 95^o^C followed by 45 cycles of 95 ^o^C for 15 min, 55^o^C for 30 sec, and 72^o^C for 30 sec. |
| Actin | Two min at 95^o^C followed by 45 cycles of 95 ^o^C for 15 min, 55^o^C for 30 sec, and 72^o^C for 30 sec. |
| GAPDH | Two min at 95^o^C followed by 45 cycles of 95 ^o^C for 15 min, 55^o^C for 30 sec, and 72^o^C for 30 sec. |
